# Clonality, latency and integration of HTLV-1 *in vivo*

**DOI:** 10.1186/1742-4690-10-S1-O17

**Published:** 2013-09-19

**Authors:** Charles Bangham, Lucy Cook, Daniel Laydon, Becca Asquith, Anat Melamed

**Affiliations:** 1 Department of Medicine, Imperial College, London, UK

## Background

The HTLV-1 proviral load set point is the strongest correlate of the inflammatory and malignant diseases associated with HTLV-1. This set point appears to be determined by an equilibrium between virus-driven proliferation and CTL-mediated killing of HTLV-1-infected T cells. However, we do not know what determines the number, the abundance or the pathogenic potential of HTLV-1-infected T cell clones. In addition, the contribution of *de novo* infection to HTLV-1 persistence in the host remains uncertain.

We hypothesize that the genomic integration site (IS) of the HTLV-1 provirus determines the pattern and intensity of spontaneous proviral expression; the viral gene products in turn determine the rate of proliferation of the infected cells, and the rate of CTL-mediated killing. We aim to identify the factors that determine the integration site targeting, expression and abundance of the HTLV-1 provirus in natural infection.

## Materials and methods

We developed a novel protocol [1,2] for high-throughput mapping and accurate quantification of proviral integration sites in the host genome in fresh uncultured peripheral blood mononuclear cells from individuals with different clinical manifestations of HTLV-1 infection.

## Results

Each infected individual carries about 25,000 distinct clones of HTLV-1-infected T cells - between 100 and 1000 times more than previously believed.

HTLV-1 preferentially integrates into host DNA within 10 to 100 bases of binding sites for specific DNA-binding factors, notably P53 and STAT1. Spontaneous expression of HTLV-1 Tax is associated with antisense orientation of the provirus in the host gene and with specific transcription factor binding sites upstream or downstream of the provirus. Genomic hotspots of HTLV-1 integration are observed in cases of adult T-cell leukaemia/lymphoma (ATLL).

HTLV-1 preferentially survives *in vivo* when integrated in an acrocentric chromosome.

## Conclusions

The results suggest that transcriptional interference and chromatin remodelling are critical determinants of proviral latency in natural HTLV-1 infection. These results open the way to tests of the molecular mechanisms of targeting, expression and clone proliferation *in vivo*.
